# Novel *CLCN4* variant associated with syndromic X-linked intellectual disability in a Chinese girl: a case report

**DOI:** 10.1186/s12887-021-02860-4

**Published:** 2021-09-03

**Authors:** Xin Xu, Fen Lu, Li Zhang, Hongying Li, Senjie Du, Jian Tang

**Affiliations:** Department of Rehabilitation, Children’s Hospital of Nanjing Medical University, No. 72 Guangzhou Road, Nanjing, 210008 Jiangsu Province China

**Keywords:** X-linked syndromic mental retardation, *CLCN4* gene, Exome sequencing, Variants, Case report

## Abstract

**Background:**

The Raynaud-Claes type of X-linked syndromic mental retardation (MRXSRC) is a very rare condition, by intellectual disability ranged from borderline to profound, impaired language development, brain abnormalities, facial dysmorphisms and seizures. MRXSRC is caused by variants in *CLCN4* which encodes the 2Cl^−^/H^+^ exchanger ClC-4 prominently expressed in brain.

**Case presentation:**

We present a 3-year-old Chinese girl with intellectual disability, dysmorphic features, brain abnormalities, significant language impairment and autistic features. Brain magnetic resonance imaging (MRI) showed a thin corpus callosum, a mega cisterna magna and ventriculomegaly. Whole exome sequencing (WES) was performed to detect the molecular basis of the disease. It was confirmed that this girl carried a novel heterozygous missense variant (c.1343C > T, p.Ala448Val) of *CLCN4* gene, inherited from her mother. This variant has not been registered in public databases and was predicted to be pathogenic by multiple in silico prediction tools.

**Conclusion:**

Our investigation expands the phenotype spectrum for *CLCN4* variants with syndromic X-linked intellectual disability, which help to improve the understanding of *CLCN4*-related intellectual disability and will help in genetic counselling for this family.

**Supplementary Information:**

The online version contains supplementary material available at 10.1186/s12887-021-02860-4.

## Introduction

The Raynaud-Claes type of X-linked syndromic mental retardation (MRXSRC, OMIM: 300114) is a rare X-linked intellectual developmental disorder characterized by borderline to severe intellectual disability (ID) and impaired language development. Additional features include brain abnormalities, facial dysmorphisms, seizures, behavioral problems, psychiatric disorders, and progressive ataxia [1–3]. Intellectual disability of the affected males was variable, even within families. Some heterozygous females are unaffected, whereas others are affected with a severity spectrum similar to that seen in males. MRXSRC is caused by heterozygous loss-of-function variants in *CLCN4* (OMIM: 302910) on chromosome Xp22.2 [1, 2]. *CLCN4* encodes the 2Cl^−^/H^+^ exchanger ClC-4, which is a member of the chloride channel (CLC) family and most homologous to ClC-3 and ClC-5 [4, 5]. Like its homologues, ClC-4 is a strongly outwardly rectifying 2Cl−/H+ exchanger and predominantly resides on intracellular vesicles [6]. Variable neurological disorders such as neurodegeneration, leukodystrophy, mental retardation, myotonia have been described in humans and mice with variants in other members of the CLC protein family [5]. The biological function of ClC-4 may be related to the ion homeostasis, including endosomal acidification and trafficking [7, 8]. However, the actual physiological function of ClC-4, particularly in the brain, remains unclear. Variants in *CLCN4* was first dentified in five families with variable X-linked intellectual disability and seizure disorder in 2016 [1]. To date, only about 40 patients with 22 variants were reported in Human Gene Mutation Database (HGMD) and literature [1–3, 9–12]. Herein, we report a novel heterozygous variant of *CLCN4* in a Chinese girl diagnosed with MRXSRC and the corresponding phenotypes.

## Case presentation

**Fig. 1:**
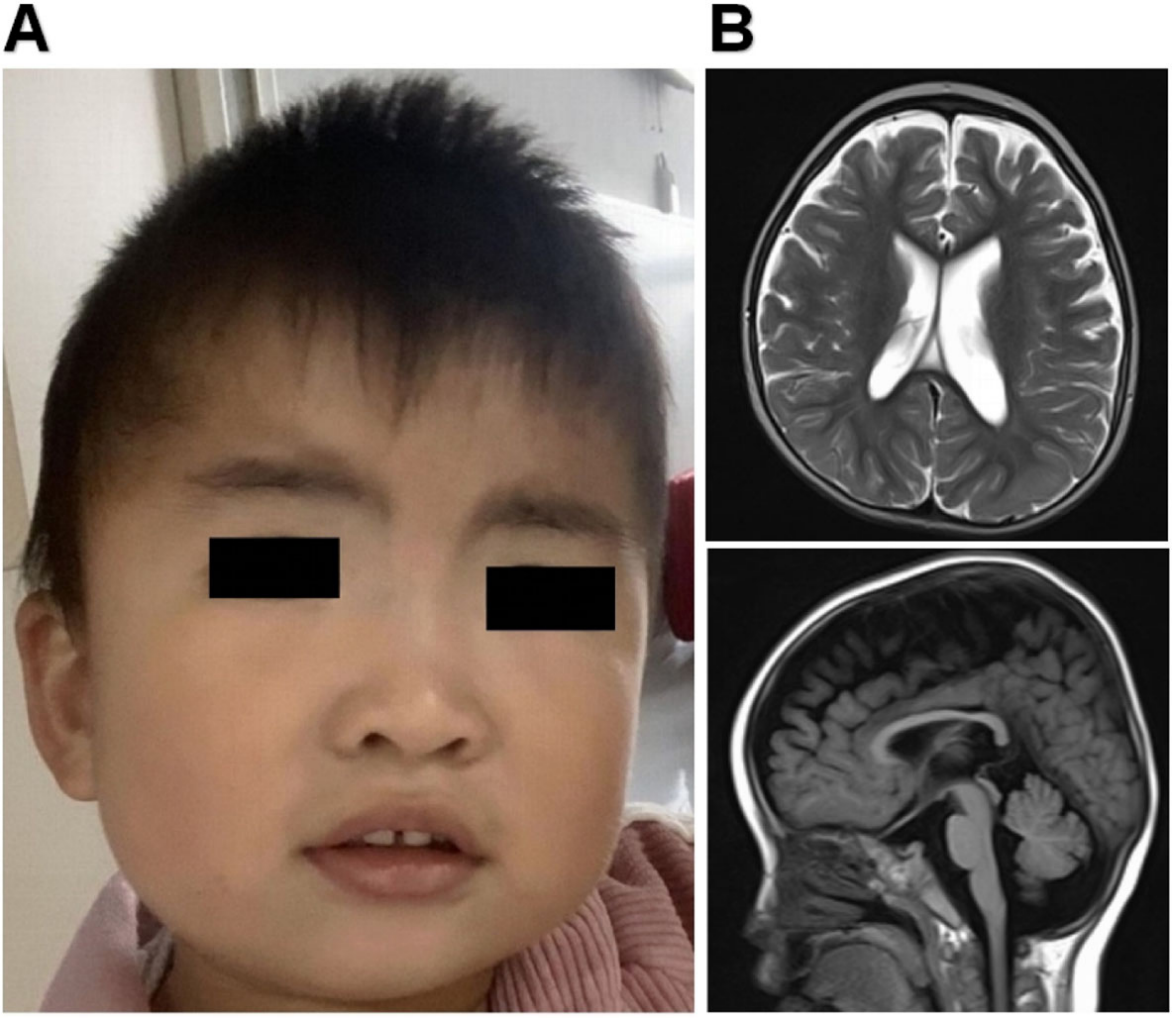
Clinical and imaging features of the proband. **a** The picture illustrates the facial dysmorphic features consisting of bushy eyebrows, downslanted palpebral fissures, esotropia, depressed nasal bridge, and sparse teeth. **b** Brain MRI showed a thin corpus callosum, a mega cisterna magna and ventriculomegaly when the girl was 3 years old

**Fig. 2:**
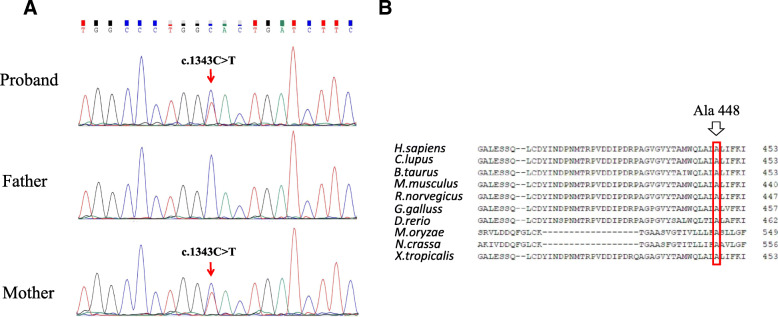
Sequencing results and analysis. **a** The variation of c.1343C > T, p.Ala448Val is a missense variant identified in the proband and her mother. Her father did not carry the variant. Red arrows indicate the variant. **b** Orthologous protein sequence alignment of *CLCN4* from different species. The mutated residue demonstrating conservation of alanine (Ala) at codon 448 is indicated by the arrow

The proband is a 3-year-old female who visited our Department of Rehabilitation for delayed language development. She was the first child of non-consanguineous Chinese parents. Her antenatal and birth history were unremarkable. She was born by cesarean section with weight of 3.2 kg and length of 50 cm. She presented hypotonia at 6 months old. The girl was able to sit unsupported around the age of 10 months and walk independently at 28 months. At the age of 3 years, she received a comprehensive physical examination. Her height was 95 cm (50th–75th percentile), weight was 15 kg (50th–75th percentile) and head circumference was 47.5 cm (10th–25th percentile). Her dysmorphic features included bushy eyebrows, downslanted palpebral fissures, esotropia, depressed nasal bridge, and sparse teeth (Fig. 1a). She had significant speech delay and did not produce any meaningful words. She showed poor eye contact and was not interested in her surroundings, so she had an abnormal social interaction. Her blood counts, liver and renal function tests, thyroid profile, metabolic screen by mass spectrometry were normal. Brain magnetic resonance imaging (MRI) showed a thin corpus callosum, a mega cisterna magna and ventriculomegaly (Fig. 2b). Long term video-Electroencephalography (EEG) showed that atypical sharp waves were emitted in the right occipital and posterior temporal regions, but she had no history of seizures. Ophthalmological and hearing assessment were normal. According to the Gesell Developmental Diagnostic Scale for children, the proband’s delayed speech indicated a developmental age of only 8 months. She was also evaluated by Autistic Behavior Checklist (ABC) and Childhood Autism Rating Scale (CARS) (ABC: score 61; CARS: score 36). A detailed study of the family history did not show any relatives with a similar presentation. Only the proband’s mother had mild symptoms. Her mother graduated from middle school with poor grades. Her pronunciation was not clear and the language expression ability was slightly poor. And the intelligence test score is 85.

To identify the underlying disease, the whole exome sequencing was performed. The study protocol was approved by the Ethics Committee of Children’s Hospital of Nanjing Medical University. Written informed consent was obtained from the proband’s parents for the publication of any potentially identifiable images or data included in this article. The method of WES was mentioned in the previous report [13]. All genetic variants were screened and listed in Table S1: Additional file 1. Finally, the candidate variants were evaluated using the American College of Medical Genetics and Genomics (ACMG) guidelines criteria [14]. Findings from WES were confirmed by Sanger sequencing in the trio. The primer sequences for the variant to be confirmed were forward 5′-GTCTTCCCAGCTCTGTGACT-3′ and reverse 5′- TGTGAAGTGGGTTTGATGC-3′. We identified the proband carried a heterozygous missense variant c.1343C > T, p.Ala448Val in exon 9 of *CLCN4* gene (Genbank association number NM_001830). Sanger sequencing was applied to confirm the variant (Fig. 2a). Her mother was confirmed to be the carrier of the heterozygous variant. The mother is a symptomatic carrier with a milder clinical phenotype. The variant co-segregation is consistent to the X-linked hereditary mode in genetics. This missense variant causes an amino acid substitution of an alanine residue with a valine residue (p.Ala448Val). Comparative amino acid sequence alignment of *CLCN4* across different species at https://www.ncbi.nlm.nih.gov/homologene revealed that the alanine at position 448 is highly conserved (Fig. 2b). The identified variant was not found in searched public databases (ExAC, gnomAD, dbSNP and 1000 Genomes Project). This variant was predicted by SIFT、PolyPhen-2、MutationTaster、GERP++ and REVEL, and the results were all damaging (Table 1). This suggested that this missense variant could be responsible for XLID in this girl.

**Table 1:** Evaluation of possible impact of c.1343C > T, p.Ala448Val variant of *CLCN4* by different bioinformatic prediction tools

Variant	SIFT score	PolyPhen-2	MutationTaster	GERP++	REVEL
c.1343C > T,	0.003	0.868	1	5.44	0.856
p.Ala448Val	Damaging	Possibly damaging	Disease causing	Damaging	Damaging

## Discussion and conclusions

X-linked intellectual disability (XLID) is a clinically and genetically heterogeneous disorder [15, 16]. To date, there are more than 100 genes associated with XLID [17]. MRXSRC, which is caused by variants in the *CLCN4* gene, is considered as a syndromic form of X-chromosome-linked ID, behavioral disorder, brain abnormalities, facial dysmorphisms and seizures. The variants in *CLCN4* result in complete penetrance but variable expressivity in males, and incomplete penetrance and variable expressivity in females. The variability of symptoms in females is not correlated with the X inactivation pattern studied in their blood [1, 2]. Palmer et al. summarized 5 heterozygous females with de novo mutations in *CLCN4* who had a more severe phenotype consistent with the phenotype in hemizygous males [2]. One had borderline, 2 had moderate, and 2 had severe/profound intellectual disability. All 5 had impaired language development. Two girls had seizure disorders of varying severity, 2 had self-injurious behaviors, and 1 was assessed as emotionally reactive. In our study, the proband with the novel variant of *CLCN4* (c.1343C > T, p.Ala448Val) displayed delayed intellectual development, lack of language expression ability, autistic features, dysmorphic features and brain abnormalities, which are consistent with the characteristic clinical manifestations of MRXSRC. Although the proband had no history of seizures, but the video-EEG showed that she had epileptiform discharges. So the proband still have the risk of seizures and need further follow-up. In this family, the mother was carrier for the variant, but her clinical symptoms were mild.

The *CLCN4* gene, which is mapped to chromosome Xp22.2, consists of 13 exons and encodes a 760 amino acids protein ClC-4. ClC-4 proteins form homodimers with a separate ion pathway within each subunit [18]. Each subunit is composed of 10 helical-transmembrane domains, 6 helical-intramembrane domains and cytosolic cystathionine-beta-synthase (CBS) domains. Transmembrane domains form the ion pathway while CBS domains affect membrane localization and regulate the transmembrane component [4]. ClC-4 is significantly expressed in the brain, especially in pyramidal cells, dentate gyrus of the hippocampus and the cerebellum Purkinje cell layer [19, 20]. Until now, the actual physiological role of ClC-4 is unclear. Previous studies had found that knockdown of the Clcn4 gene in mouse hippocampal neurons resulted in 30% less dendritic branches compared to controls, and cultured neurons derived from Clcn4^−/−^mice showed similar changes [1, 20, 21]. This indicates that ClC-4 play a important role in the development of the nervous system.

**Fig. 3:**
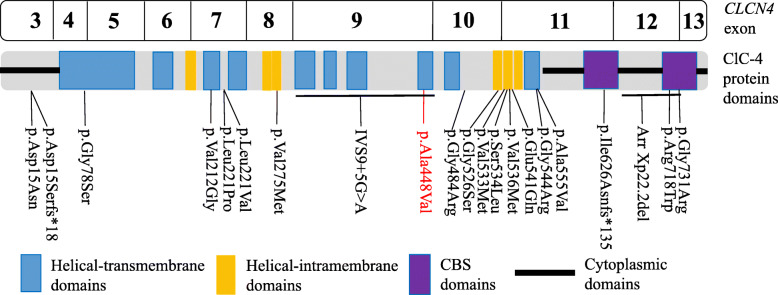
Schematic diagrams showing structure of ClC-4. The variants were reported in HGMD and the literatures were shown in black, respectively. The position of the variant identified in this study are shown in red. CBS, cytosolic cystathionine-beta-synthase

To date, there are eighteen missense variants, two frameshift variants, one splice site variant, one exonic deletion variant in *CLCN4* reported in HGMD and the literatures (Fig. 3). As to our patient, She was found to have a novel missense variant (c.1343C > T, p.Ala448Val) in *CLCN4* which is located in the eighth helical transmembrane domain region of exon 9. As reported, the majority of the missense variants affect residues in the transmembrane or intramembrane part that contains the ion translocation pathway, often close to the interface of the two subunits of the ClC-4 protein [2, 20]. And these variants reduced or abolished the outwardly rectifying ClC-4 exchange currents in heterologous expression in vitro [1, 2, 20]. Our variant causes changes in conserved amino acids, which are predicted to be pathogenic by multiple in silico prediction tools, suggesting the pathogenicity of this variant. However, in vitro functional expression studies are needed for further verification. The intronic splice site variant (IVS9 + 5G > A) in a male patient with severe intellectual disability reported in the literature was verified to affect splicing leading to in-frame deletion of exon 9 [2]. Therefore, exon 9 is predicted to be a critical exon as it codes for four helical transmembrane domains.

In conclusion, in this study we found a novel heterozygous missense variant of XLID-related *CLCN4* gene. Through the analysis of clinical manifestations and the mutated gene of the girl, it was speculated that the likely cause of XLID in this case is a missense variant in *CLCN4.* At present, the understanding of the molecular mechanism of XLID is not comprehensive, and the findings of this study expand the expression spectrum and gene spectrum of XLID, which can provide a reference for family counseling.

## Supplementary Information

**Additional file 1: Table S1.** Workflow of filtering the pathogenitic variant.

## Data Availability

The datasets used and/or analyzed during the current study are available from the corresponding author on reasonable request.

## Abbreviations

MRXSRC: The Raynaud-Claes type of X-linked syndromic mental retardation
OMIM: Online Mendelian Inheritance in Man
WES: Whole exome sequencing
ID: Intellectual disability
HGMD: Human Gene Mutation Database
CLC: Chloride channel
MRI: Brain magnetic resonance imaging
EEG: Electroencephalogram
ABC: Autistic Behavior Checklist
CARS: Childhood Autism Rating Scale
ACMG: American College of Medical Genetics and Genomics
XLID: X-linked intellectual disability
CBS: cystathionine-beta-synthase

## References

[1] Hu H, Haas SA, Chelly J, Van Esch H, Raynaud M, de Brouwer AP (2016). X-exome sequencing of 405 unresolved families identifies seven novel intellectual disability genes. Mol Psychiatry.

[2] Palmer EE, Stuhlmann T, Weinert S, Haan E, Van Esch H, Holvoet M (2018). De novo and inherited mutations in the X-linked gene *CLCN4* are associated with syndromic intellectual disability and behavior and seizure disorders in males and females. Mol Psychiatry.

[3] Veeramah KR, Johnstone L, Karafet TM, Wolf D, Sprissler R, Salogiannis J (2013). Exome sequencing reveals new causal mutations in children with epileptic encephalopathies. Epilepsia..

[4] Scheel O, Zdebik AA, Lourdel S, Jentsch TJ (2005). Voltage-dependent electrogenic chloride/proton exchange by endosomal CLC proteins. Nature..

[5] Jentsch TJ (2015). Discovery of CLC transport proteins: cloning, structure, function and pathophysiology. J Physiol.

[6] Picollo A, Pusch M (2005). Chloride/proton antiporter activity of mammalian CLC proteins ClC-4 and ClC-5. Nature..

[7] Mohammad-Panah R, Harrison R, Dhani S, Ackerley C, Huan LJ, Wang Y (2003). The chloride channel ClC-4 contributes to endosomal acidification and trafficking. J Biol Chem.

[8] Suzuki T, Rai T, Hayama A, Sohara E, Suda S, Itoh T (2006). Intracellular localization of ClC chloride channels and their ability to form hetero-oligomers. J Cell Physiol.

[9] Snoeijen-Schouwenaars FM, van Ool JS, Verhoeven JS, van Mierlo P, Braakman HMH, Smeets EE (2019). Diagnostic exome sequencing in 100 consecutive patients with both epilepsy and intellectual disability. Epilepsia..

[10] Fernández-Marmiesse A, Roca I, Díaz-Flores F, Cantarín V, Pérez-Poyato MS, Fontalba A (2019). Rare variants in 48 genes account for 42% of cases of epilepsy with or without neurodevelopmental delay in 246 pediatric patients. Front Neurosci.

[11] Kosmicki JA, Samocha KE, Howrigan DP, Sanders SJ, Slowikowski K, Lek M (2017). Refining the role of de novo protein-truncating variants in neurodevelopmental disorders by using population reference samples. Nat Genet.

[12] Retterer K, Juusola J, Cho MT, Vitazka P, Millan F, Gibellini F (2016). Clinical application of whole-exome sequencing across clinical indications. Genet Med..

[13] Wang C, Han Y, Zhou J, Zheng B, Zhou W, Bao H (2020). Splicing characterization of CLCNKB variants in four patients with type III Bartter syndrome. Front Genet.

[14] Richards S, Aziz N, Bale S, Bick D, Das S, Gastier-Foster J (2015). Standards and guidelines for the interpretation of sequence variants: a joint consensus recommendation of the American College of Medical Genetics and Genomics and the Association for Molecular Pathology. Genet Med.

[15] de Brouwer AP, Yntema HG, Kleefstra T, Lugtenberg D, Oudakker AR, de Vries BB (2007). Mutation frequencies of X-linked mental retardation genes in families from the EuroMRX consortium. Hum Mutat.

[16] van Bokhoven H (2011). Genetic and epigenetic networks in intellectual disabilities. Annu Rev Genet.

[17] Neri G, Schwartz CE, Lubs HA, Stevenson RE (2018). X-linked intellectual disability update 2017. Am J Med Genet A.

[18] Feng L, Campbell EB, Hsiung Y, MacKinnon R (2010). Structure of a eukaryotic CLC transporter defines an intermediate state in the transport cycle. Science..

[19] Park E, Campbell EB, MacKinnon R (2017). Structure of a CLC chloride ion channel by cryo-electron microscopy. Nature..

[20] Jentsch TJ, Pusch M (2018). CLC chloride channels and transporters: structure, function, physiology, and disease. Physiol Rev.

[21] Hur J, Jeong HJ, Park J, Jeon S (2013). Chloride channel 4 is required for nerve growth factor-induced TrkA signaling and neurite outgrowth in PC12 cells and cortical neurons. Neuroscience..

